# Impact of the Micropapillary Variant in Urothelial Carcinoma of the Bladder—A Comprehensive Review of Meta-Analyses and Contemporary Cohort Studies

**DOI:** 10.3390/cancers18050727

**Published:** 2026-02-24

**Authors:** Agnieszka Leśniewska-Bocianowska, Jan Bocianowski

**Affiliations:** 1 Department of Pathophysiology of Ageing and Civilization Diseases, Poznan University of Medical Sciences, Święcickiego 4, 60-781 Poznań, Poland; 2 Department of Mathematical and Statistical Methods, Poznań University of Life Sciences, Wojska Polskiego 28, 60-637 Poznań, Poland

**Keywords:** urinary bladder cancer, micropapillary urothelial carcinoma, histological variants, prognostic factors, radical cystectomy, neoadjuvant chemotherapy, upstaging

## Abstract

**Background/Objectives**: Micropapillary urothelial carcinoma of the urinary bladder (MPUC) represents a rare but highly aggressive histological variant of urothelial carcinoma (UC), frequently presenting at an advanced stage of disease. Although data on histological variants consistently suggest inferior oncological outcomes, the independent prognostic role of the micropapillary variant remains controversial. **Methods**: The present work synthesizes the findings of a large meta-analysis evaluating histological variants of UC and a separate meta-analysis focusing exclusively on MPUC, and further examines the most recent cohort-based evidence published between 2016 and 2025. **Results**: The presence of any histological variant in UC treated with radical cystectomy is associated with significantly worse recurrence-free survival, disease-specific survival, and overall survival, as reflected by pooled hazard ratios (HRs). For the micropapillary variant specifically, a modest increase in overall mortality has been demonstrated (pooled HR ≈ 1.20); however, results from adjusted analyses dedicated to MPUC remain inconsistent. **Conclusions**: Micropapillary urothelial carcinoma is consistently associated with adverse pathological features and poorer oncological outcomes. However, whether micropapillary morphology independently predicts prognosis beyond established factors such as pathological stage and nodal status remains uncertain, as adjusted analyses across studies have yielded inconsistent results. Part of the observed survival disadvantage may be explained by stage migration, although an intrinsic residual risk cannot be definitively ruled out. This review integrates contemporary population-based registry analyses with prior meta-analytic evidence to provide a clinically oriented synthesis of the prognostic and therapeutic implications of MPUC. In addition, we propose minimal reporting standards aimed at improving comparability across future studies and strengthening risk stratification and treatment decision-making.

## Simple Summary

Micropapillary urothelial carcinoma of the bladder is a rare but highly aggressive form of bladder cancer. It is often diagnosed at an advanced stage and is associated with early disease progression and frequent lymph node metastases. This review summarizes evidence from meta-analyses and recent cohort studies published between 2016 and 2025. The available data consistently show worse survival outcomes compared with conventional urothelial carcinoma, largely due to frequent understaging and advanced pathological features at diagnosis. Bladder-preserving treatment is often ineffective, particularly in high-grade non-muscle-invasive disease, supporting the use of early radical cystectomy. Although micropapillary tumors may respond to chemotherapy, this response does not fully offset their aggressive biological behavior. Better recognition and standardized reporting of this variant may improve treatment decisions and patient outcomes.

## 1. Introduction

Urothelial carcinoma of the urinary bladder comprises, in addition to the conventional form, several distinct histological variants, including squamous, glandular, plasmacytoid, sarcomatoid, and micropapillary forms [1,2,3,4,5,6]. Among these, micropapillary urothelial carcinoma (MPUC) is relatively rare—accounting for approximately 1–5% of all bladder malignancies—yet is associated with a disproportionately poor clinical prognosis [7]. Since its initial description, MPUC has been increasingly identified in routine pathological practice, leading to greater awareness of its clinical relevance [8].

MPUC of the urinary bladder represents a rare but clinically significant histological subtype characterized by aggressive behavior, a high risk of early disease progression, and unfavorable oncological outcomes [9]. Despite its low reported incidence, typically estimated at 0.7–2.2% of all bladder cancers [10], MPUC carries substantial prognostic and therapeutic implications, particularly with regard to decisions surrounding early radical treatment strategies (Table 1).

The micropapillary variant of urothelial carcinoma was first described in the urinary bladder in the 1990s and remains an uncommon histological subtype [7,23]. Patients with MPUC frequently present with advanced-stage disease, early lymph node involvement, and a pronounced risk of progression. Importantly, even focal presence of a micropapillary component within an otherwise conventional urothelial carcinoma may be associated with an aggressive clinical course [9,23]. These traits pose significant clinical challenges and raise critical questions concerning optimal diagnostic approaches, risk stratification, and treatment selection in patients with variant histology of urothelial carcinoma, as variant morphology may influence both prognosis and therapeutic strategy [24].

From a clinical perspective, MPUC is particularly concerning because many cases are diagnosed at an advanced stage and demonstrate lymph node metastases at the time of radical cystectomy [25]. This pattern suggests a more aggressive biological behavior compared with classical urothelial carcinoma. Consequently, some authors advocate for intensified treatment strategies, such as early radical cystectomy, even in scenarios where standard criteria might otherwise support bladder-preserving approaches.

The rarity of MPUC, together with substantial heterogeneity in reporting practices—including variability in the proportion and extent of the micropapillary component and inconsistent use of perioperative chemotherapy—complicates data interpretation and limits the generalizability of published findings. Two meta-analyses [11,26]—one encompassing a broad spectrum of histological variants and the other focused exclusively on MPUC—have provided valuable yet partially conflicting evidence. In this short communication, these data are summarized and critically appraised alongside the most recent cohort studies published between 2016 and 2025, with the aim of better informing clinical practice and identifying priorities for future research. The objective of this review is to deliver a comprehensive synthesis of high-quality available evidence addressing the prognostic impact, therapeutic implications, and biological characteristics of MPUC.

## 2. Materials and Methods

### 2.1. Study Design—Inclusion and Exclusion Criteria

This systematic review was conducted in accordance with the Preferred Reporting Items for Systematic Reviews and Meta-Analyses (PRISMA) guidelines [27]. This review was not registered; nevertheless, the study followed a prespecified protocol and was conducted in accordance with PRISMA guidelines. A comprehensive and structured search of the scientific literature was conducted using the PubMed, Embase and Scopus databases. A comprehensive search strategy was developed using a combination of controlled vocabulary and free-text keywords. The primary search terms included: “micropapillary bladder cancer” or “carcinoma” or “micropapillary urothelial cancer” or “micropapillary urothelial carcinoma” or “micropapillary histological variant” or “prognosis”.

Inclusion/exclusion criteria: The review was restricted to bladder MPUC; however, one study investigating the upper urinary tract variant was retained due to its relevance to molecular alterations and potential therapeutic targets shared across urothelial carcinomas. This study was not used to support bladder-specific clinical conclusions.

### 2.2. Data Extraction

A structured literature review was conducted based on 12 key publications [11,12,13,14,15,16,17,18,19,20,21,22]. From the meta-analysis of histological variants, pooled hazard ratios (HRs) were extracted for recurrence-free survival (RFS), disease-specific survival (CSS), and overall survival (OS), as well as HRs specific to the micropapillary subgroup. From the meta-analysis focusing on MPUC, qualitative conclusions were summarized regarding disease stage at diagnosis, treatment response, and survival outcomes according to the therapeutic approach applied (e.g., early radical cystectomy vs. TURBT plus BCG).

In addition, more recent cohort and database studies published after 2016 were reviewed to capture evolving trends, variability in clinical practice, and outcomes in real-world settings. Particular attention was paid to key methodological sources of heterogeneity, including: (1) adjustment for disease stage and nodal status in statistical analyses; (2) reporting and quantitative assessment of the micropapillary component; (3) use and timing of perioperative therapy; and (4) the choice of oncological endpoints.

The average values for cT1, cT2, cT3, and cT4 as well as the average percentages of estimated risk of progression for BCG-based bladder presentation and upfront radical cystectomy as well as the average percentages of pathological complete response for RC alone and NAC + RC are simple average values calculated based on data contained in basic publications [11,12,13,14,15,16,17,18,19,20,21,22]. The relationship between the estimated upstaging rate and clinical T stage was evaluated using linear regression analysis. The coefficient of determination (*R*^2^) was used as a measure of goodness of fit. All statistical analyses were performed using Genstat software, version 24.2 [28]. In addition, the methodological quality of the two principal meta-analyses included in this synthesis [11,26] was critically appraised. Particular attention was paid to predefined protocol registration, comprehensiveness of literature search, handling of heterogeneity, assessment of publication bias, and adjustment for confounding variables in the primary studies. Although a formal AMSTAR-2 scoring was not conducted due to the narrative design of the present review, key domains relevant to risk of bias were qualitatively evaluated to contextualize the strength of pooled estimates.

## 3. Study Selection

**Figure 1:**
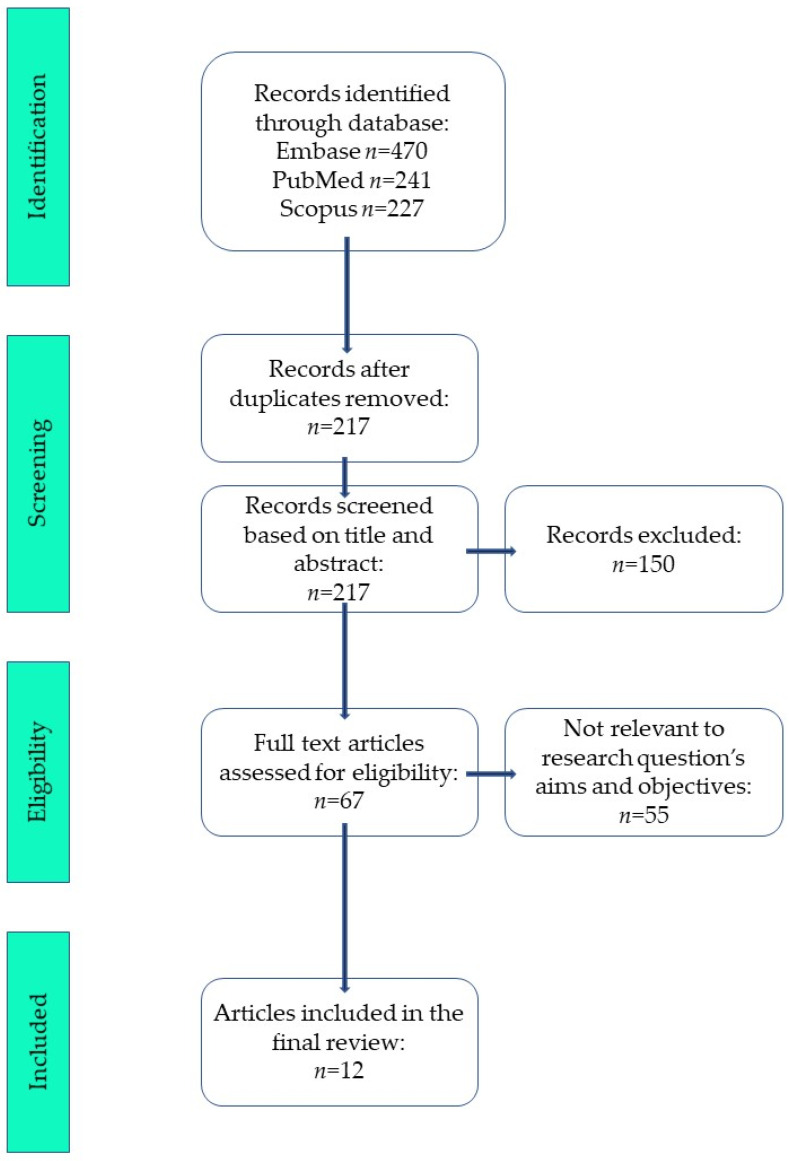
Flow diagram of the study selection process based on PRISMA 2020 [27] guidelines.

The database search identified a total of 938 records (Embase *n* = 470, PubMed *n* = 241, Scopus *n* = 227). After removal of duplicates, 217 records remained and were screened based on title and abstract. Of these, 150 records were excluded. Sixty-seven full-text articles were assessed for eligibility, and 55 were excluded as not relevant to the research question and objectives. Finally, 12 articles were included in the review. The PRISMA flow diagram summarizing the study selection process is presented in Figure 1.

## 4. Prognostic Significance: Synthesis of Evidence from Meta-Analyses and Cohort Studies

The most comprehensive assessment of the prognostic impact of MPUC was provided by Abufaraj et al. [11] in a meta-analysis including more than 1400 patients. The authors demonstrated significantly worse overall survival (OS) and cancer-specific survival (CSS) in patients with MPUC compared with those with conventional urothelial carcinoma, both in organ-confined disease and in locally advanced stages. The micropapillary variant was also associated with a higher likelihood of advanced pathological stage (≥pT3), lymph node metastases, and positive surgical margins.

Findings from large population-based analyses, including data from the National Cancer Database (NCDB), corroborate these observations. Sui et al. [12] reported that even a minor micropapillary component correlated with inferior survival outcomes, with patients more frequently presenting with high-grade and advanced-stage disease. Similar conclusions were drawn by Mitra et al. [13] and Jin et al. [14], both of whom identified MPUC as an independent risk factor for reduced OS and CSS following radical cystectomy.

More recent cohort studies, including those by Chou et al. [17] and Jojima et al. [18], have confirmed high rates of lymph node involvement (30–50%) and early disease recurrence, even among patients with clinically NMIBC. Predictive models and nomograms proposed by Liu et al. [19] further indicate that the proportion of the micropapillary component, pathological T stage, and nodal status represent some of the strongest predictors of survival outcomes in this patient population. These clinicopathological observations provide the foundation for therapeutic decision-making and explain why treatment strategies in MPUC often diverge from standard algorithms applied to conventional urothelial carcinoma.

## 5. Therapeutic Implications

### 5.1. Local Treatment and Decisions Regarding Early Radical Cystectomy

Given the consistent association of MPUC with advanced pathological features and high rates of upstaging, treatment strategies must account for its aggressive clinical phenotype. Accumulating evidence consistently suggests that standard bladder-preserving management with transurethral resection of the bladder tumor (TURBT) followed by intravesical bacillus Calmette–Guérin (BCG) immunotherapy is insufficient for a substantial proportion of patients with MPUC, particularly those with high-grade T1 disease. The meta-analysis by Abufaraj et al. [11], together with multiple cohort studies, demonstrated a high risk of progression to MIBC among patients managed conservatively. Consequently, an increasing number of authors [13,17,20] advocate for early radical cystectomy in patients with high-risk NMIBC MPUC, even when the micropapillary component represents only a minor proportion of the tumor. The discrepancy between clinical and pathological staging plays a pivotal role in therapeutic decision-making in MPUC. Given the consistently reported high rates of upstaging from cT1–cT2 to ≥pT3 disease at the time of radical cystectomy [11,12,18], reliance on clinical staging alone may result in undertreatment if bladder-preserving strategies are selected. This staging discordance should therefore be explicitly incorporated into individualized risk assessment. In patients with high-grade cT1 MPUC, particularly those with a substantial micropapillary component, lymphovascular invasion, or radiographic suspicion of nodal involvement, early radical cystectomy should be strongly considered despite apparent NMIBC status [12,17,23]. Conversely, in carefully selected patients with limited micropapillary differentiation, absence of adverse pathological features, and reliable restaging evaluation, a bladder-preserving approach may be discussed within a multidisciplinary framework, provided that close surveillance is ensured. For MIBC, treatment planning should integrate clinical stage, radiographic nodal assessment, performance status, and estimated risk of occult nodal metastasis. While neoadjuvant chemotherapy may achieve pathological downstaging in selected cases, its use should not delay definitive surgical management in patients at high risk of pathological upstaging [13,15,16]. Overall, management of MPUC requires individualized decision-making that explicitly accounts for the high probability of clinical understaging and the biological aggressiveness of this variant.

### 5.2. Neoadjuvant Chemotherapy (NAC)

The role of neoadjuvant chemotherapy (NAC) in MPUC remains a matter of ongoing debate. Studies by Diamantopoulos et al. [15] and Rahman et al. [16] indicate that pathological response rates to NAC in MPUC are comparable to those observed in conventional urothelial carcinoma, including the achievement of pathological complete response (pCR). However, the associated survival benefit appears less consistent (Table 2). Several analyses suggest that the intrinsically aggressive biology of MPUC may limit the long-term benefits of NAC, particularly in patients with a substantial micropapillary component and lymph node involvement. Nevertheless, in selected patients with MIBC, NAC may improve the feasibility and outcomes of definitive surgical management (Table 2).

MPUC appears to retain sensitivity to cisplatin-based chemotherapy, as reflected by the potential to achieve pCR; however, in most published studies, NAC does not fully offset the adverse biological behavior of the tumor. The survival benefit associated with NAC is modest and likely dependent on careful patient selection, favoring individuals with a low proportion of the micropapillary component and absence of clinically apparent nodal disease (cN0). Early radical cystectomy therefore remains a cornerstone of treatment in MPUC (Table 2).

**Figure 2:**
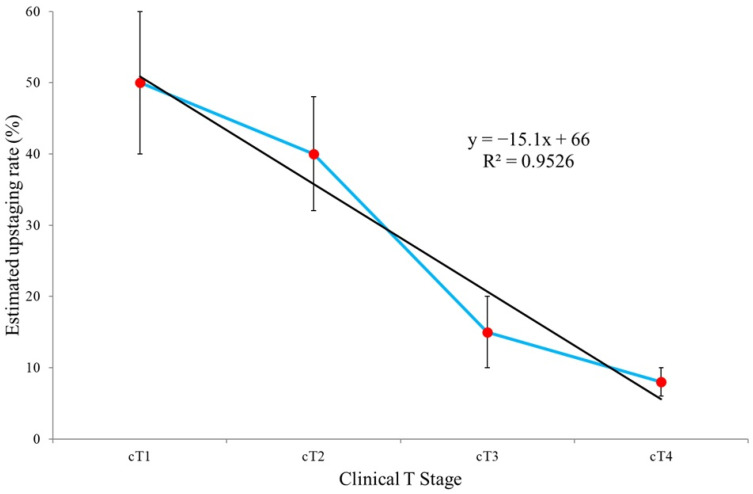
Upstaging from Clinical to Pathological T Stage in micropapillary urothelial carcinoma. The presented values for cT1, cT2, cT3, and cT4 are average values calculated based on data published in the primary publications [11,12,13,14,15,16,17,18,19,20,21,22]; bars characterize standard deviations for individual T Stages. The linear model was saved and estimated for the purposes of this study and is proprietary.

In patients with MPUC, clinical T stage frequently fails to reflect the true biological aggressiveness of the tumor, and underestimation of disease extent (upstaging) is common, particularly in cT1–cT2 disease. High upstaging rates have been observed in cT1–cT2 tumors (approximately 40–60%), underscoring the limited reliability of clinical staging in MPUC (Figure 2). Trend line analysis demonstrates a statistically significant decrease in the estimated upstaging rate across successive clinical T stages. The regression coefficient of −15.1 was statistically significant at the 0.001 level, while the coefficient of determination (*R*^2^ = 0.9526) indicates an excellent model fit to the observed data.

Importantly, even apparently early clinical stages are associated with a high risk of disease progression and lymph node metastasis, supporting aggressive therapeutic strategies regardless of clinical T stage (Table 3). Pathological T stage remains one of the strongest predictors of survival in MPUC. The high prevalence of ≥pT3 disease even among patients with low clinical T stage further confirms the limited accuracy of clinical assessment (Table 4). Overall, MPUC is characterized by an exceptionally high risk of upstaging, particularly in cT1–cT2 disease, which has critical implications for treatment planning (Table 5).

**Figure 3:**
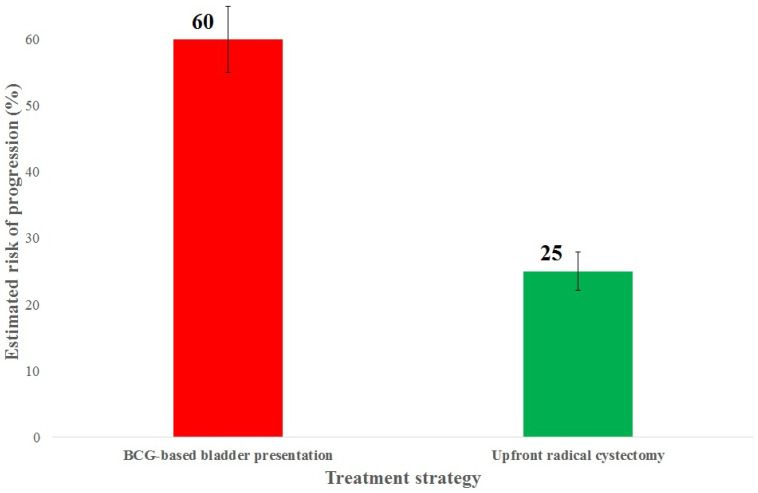
Clinical Outcomes in cT1 Micropapillary Bladder Cancer. The presented percentages of estimated risk of progression for BCG-based bladder presentation and upfront radical cystectomy are average values calculated based on data published in the primary publications [11,12,13,14,15,16,17,18,19,20,21,22]. *N* = 2881.

**Figure 4:**
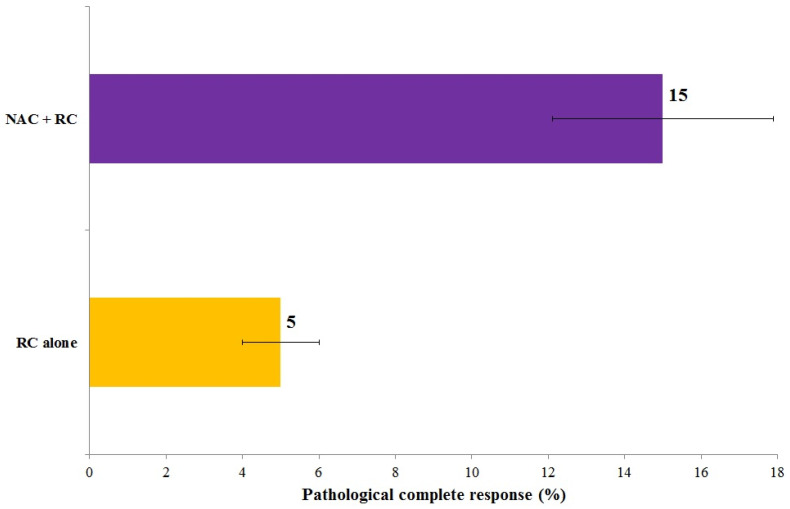
Effect of Neoadjuvant Chemotherapy in MIBC MPUC. The presented percentages of pathological complete response for RC alone and NAC + RC are average values calculated based on data published in the primary publications [11,12,13,14,15,16,17,18,19,20,21,22]. *N* = 2881.

In patients with cT1 MPUC, intravesical therapy with bacillus Calmette–Guérin (BCG) is associated with a high risk of treatment failure and disease progression. Data from meta-analyses and cohort studies consistently support an upfront radical cystectomy strategy in patients with high-risk cT1 MPUC (Table 6). Comparative outcomes of BCG-based bladder preservation versus upfront radical cystectomy are illustrated in Figure 3, demonstrating a higher risk of progression with conservative management and further supporting upfront radical cystectomy in cT1 high-grade MPUC. Although NAC was associated with an increase in pCR rates from approximately 5% to 15% (Figure 4), these findings indicate that achievement of pCR does not equate to full compensation for the aggressive biological behavior of MPUC.

### 5.3. Immune Checkpoint Inhibitors and Systemic Therapy in Advanced Disease

Immune checkpoint inhibitors (ICIs) have become an established component of systemic therapy in advanced urothelial carcinoma, including both platinum-refractory and maintenance settings. Agents targeting the PD-1/PD-L1 axis, such as pembrolizumab and avelumab, have demonstrated survival benefit in randomized phase III trials in metastatic urothelial carcinoma [29,30,31]. However, variant histologies, including micropapillary urothelial carcinoma, were underrepresented in pivotal immunotherapy trials, and variant-specific subgroup analyses are generally lacking. As a result, high-quality evidence regarding the efficacy of ICIs specifically in MPUC remains limited. Retrospective analyses and real-world data suggest that patients with variant histology may derive benefit from immunotherapy comparable to conventional urothelial carcinoma, although conclusions are constrained by small sample sizes and histological heterogeneity [32,33]. From a biological perspective, the luminal phenotype frequently observed in MPUC, together with variable PD-L1 expression and tumor mutational burden in urothelial carcinoma, provides a rationale for considering immunotherapy in advanced or metastatic settings. Nevertheless, whether micropapillary differentiation influences immunotherapy responsiveness independently of established biomarkers remains unknown. Prospective studies incorporating detailed histological subtyping are needed to clarify the role of ICIs in this population.

## 6. Biological and Molecular Features of the Micropapillary Variant

The aggressive clinical behavior and high propensity for early dissemination observed in MPUC are increasingly understood in light of its distinct molecular profile. Investigations by Guo et al. [21] and Bowden et al. [22] demonstrated that MPUC exhibits a distinct molecular profile characterized by overexpression of genes involved in cell adhesion, HER2/ERBB2 signaling, epithelial–mesenchymal transition (EMT) pathways, and angiogenesis. MPUC frequently displays a luminal phenotype, with high expression of GATA3, KRT20, and ERBB2, distinguishing it from more basal-like forms of bladder cancer.

Concurrently, molecular features promoting early lymphovascular invasion are commonly observed, correlating with the characteristic histopathological architecture of MPUC and its high incidence of lymph node metastases. Analysis of high-grade T1 disease by Bowden et al. [22] suggests that even at an early clinical stage, MPUC already harbors a molecular signature resembling that of advanced disease, thereby providing a biological rationale for aggressive therapeutic strategies.

## 7. Data Integration and Clinical Implications

A comprehensive synthesis of available meta-analyses and cohort studies consistently indicates that the micropapillary variant of bladder cancer: (1) is consistently associated with adverse pathological characteristics and inferior survival outcomes, although its independent prognostic impact beyond stage and nodal status remains uncertain; (2) is characterized by early disease progression and a high prevalence of lymph node metastasis; (3) requires a distinct therapeutic approach compared with conventional urothelial carcinoma; and (4) exhibits a characteristic aggressive molecular profile that may render it amenable to targeted therapeutic strategies, such as HER2-directed treatments.

## 8. Limitations

This review has several limitations. First, the available evidence is predominantly retrospective, which inherently increases the risk of selection bias. Second, substantial heterogeneity exists across studies with respect to patient populations, treatment strategies, and reporting of the micropapillary component. Third, the rarity of MPUC limits the feasibility of large prospective trials. Consequently, the conclusions should be interpreted with caution.

## 9. Proposed Minimal Reporting Standards for Future Studies on MPUC

Despite the growing number of retrospective cohorts and pooled analyses, interpretation of the prognostic and therapeutic impact of MPUC remains limited by substantial heterogeneity in reporting practices. To improve comparability and enable more robust meta-analytic integration, we propose the following minimal reporting standards for future studies:

**(1) Quantitative reporting of the micropapillary component.** Studies should specify the percentage of micropapillary differentiation and clearly define whether focal and predominant forms are analyzed separately.

**(2) Standardized pathological staging.** Pathological T stage and nodal status should be reported according to current TNM criteria, with explicit distinction between clinical and pathological stage to allow assessment of stage migration.

**(3) Detailed documentation of lymph node assessment.** The number of lymph nodes removed, number of positive nodes, and presence of extranodal extension should be reported.

**(4) Transparent treatment pathways.** Studies should clearly describe whether patients received TURBT + BCG, upfront radical cystectomy, neoadjuvant chemotherapy, or multimodal therapy, including timing and selection criteria.

**(5) Uniform oncological endpoints.** Overall survival, cancer-specific survival, recurrence-free survival, and pathological complete response rates should be defined consistently, with specification of follow-up duration.

**(6) Multivariable adjustment strategy.** Statistical models should clearly indicate which clinicopathological variables were included, particularly pathological stage, nodal status, lymphovascular invasion, and perioperative treatment.

Adoption of these standardized elements would substantially reduce methodological heterogeneity and allow more definitive determination of whether micropapillary morphology exerts an independent prognostic effect beyond established staging variables.

## 10. Discussion

The present synthesis not only consolidates prior meta-analytic findings but also integrates contemporary large-scale registry analyses to provide a clinically oriented interpretation of prognostic and therapeutic evidence in MPUC. First, the presence of histological variants in urothelial carcinoma treated with radical cystectomy is associated with significantly worse survival outcomes and an increased risk of disease recurrence. Second, the micropapillary variant contributes to this overall effect, with pooled evidence indicating an approximately 20% increase in the risk of death. Importantly, this effect appears to be partly mediated by the higher prevalence of advanced pathological stage and nodal involvement in MPUC, and may not consistently persist after multivariable adjustment [13,14,26]. However, it remains uncertain whether micropapillary morphology per se represents an independent prognostic factor beyond its frequent association with advanced pathological stage and lymph node involvement. The meta-analysis by Abufaraj et al. [11] highlights that, after adjustment for disease stage, survival differences are not consistently observed. Real-world data remain similarly inconclusive: registry-based studies confirm more advanced disease at diagnosis, yet survival disparities may largely reflect this stage migration rather than an intrinsic effect of histology.

The interpretation of pooled results derived from existing meta-analyses requires careful consideration of their methodological constraints. Both Abufaraj et al. [11] and Mori et al. [26] conducted systematic reviews with comprehensive literature searches and appropriate statistical pooling methods; however, the majority of included studies were retrospective cohort analyses, frequently lacking standardized pathological review and uniform adjustment for confounding variables such as pathological stage, nodal status, and perioperative treatment. Substantial clinical and methodological heterogeneity was present, particularly regarding the proportion of the micropapillary component and indications for radical cystectomy or neoadjuvant chemotherapy. These factors introduce a non-negligible risk of bias and limit the certainty with which independent prognostic effects can be inferred. Consequently, pooled hazard ratios should be interpreted as reflecting aggregated observational evidence rather than definitive causal estimates. Future umbrella reviews applying formal tools such as AMSTAR-2 may provide a more structured appraisal of the existing meta-analytic evidence.

Overall, the available evidence confirms that MPUC of the bladder remains one of the most controversial and clinically challenging histological variants of bladder cancer. Numerous retrospective studies and meta-analyses published since 2015 consistently demonstrate its aggressive clinical course; nevertheless, a definitive answer as to whether micropapillary morphology itself constitutes an independent prognostic determinant, or merely a surrogate of advanced disease at presentation, is still lacking [11,12,13].

One of the most reproducible findings across large population-based analyses and institutional series is the high prevalence of advanced pathological stage in patients with MPUC. Analysis of the National Cancer Database by Sui et al. [12] demonstrated that even a focal micropapillary component is associated with significantly higher rates of ≥pT3 disease and more frequent lymph node metastases. These observations were independently corroborated by Mitra et al. [13] and Jin et al. [14], who consistently identified pathological stage and nodal status as the strongest determinants of survival following radical cystectomy.

A central unresolved issue concerns whether micropapillary morphology constitutes an independent prognostic factor or whether its adverse impact is largely explained by stage migration and residual confounding. The consistently higher prevalence of ≥pT3 disease and lymph node metastases in MPUC raises the possibility that inferior survival outcomes primarily reflect more advanced disease at presentation rather than intrinsic biological aggressiveness. This phenomenon corresponds to a staging migration effect, whereby clinical staging underestimates the true extent of disease and subsequently amplifies apparent prognostic differences. Importantly, multivariable analyses across available studies have yielded heterogeneous results. In the meta-analysis by Abufaraj et al. [11], survival differences were attenuated or lost after adjustment for pathological stage and nodal status, suggesting that stage distribution may account for a substantial proportion of the observed risk. Similarly, population-based analyses such as those by Sui et al. [12] and Jin et al. [14] identified pathological stage and lymph node involvement as the dominant determinants of survival. In contrast, certain institutional series, including Mitra et al. [13], reported an independent adverse effect of micropapillary histology even after multivariable adjustment. These discrepancies may reflect differences in cohort composition, extent of pathological review, quantification of the micropapillary component, and use of perioperative chemotherapy. Moreover, the possibility of residual risk beyond conventional staging variables cannot be entirely excluded. Molecular studies demonstrating early activation of epithelial–mesenchymal transition pathways and frequent ERBB2 overexpression [21,22] provide biological plausibility for an intrinsic aggressive phenotype that may not be fully captured by anatomical staging alone. Taken together, current evidence suggests that the adverse prognosis associated with MPUC is driven predominantly by advanced pathological stage and nodal involvement; however, a modest residual independent effect of micropapillary differentiation remains biologically plausible but not definitively established. Prospective studies with standardized pathological reporting and comprehensive multivariable adjustment are required to clarify this relationship [23,26].

A critical clinical issue in MPUC is the high rate of clinical understaging. Multiple studies published after 2015 have shown that 40–60% of patients initially staged as cT1–cT2 experience substantial upstaging at the time of radical cystectomy [11,12,17]. This phenomenon undermines the reliability of conventional clinical staging in MPUC and represents one of the principal arguments supporting early radical cystectomy, even in patients who formally meet criteria for high-risk NMIBC.

An important yet unresolved issue concerns the quantitative significance of the micropapillary component. Some authors suggest that even focal MPUC confers adverse prognostic implications [12,14], whereas others report a stepwise increase in risk with a growing proportion of micropapillary differentiation within the tumor [19]. The lack of standardized reporting of the percentage and extent of the micropapillary component, together with variability in pathological practice, substantially increases heterogeneity across studies and limits the ability to draw definitive conclusions.

The role of neoadjuvant chemotherapy (NAC) in MPUC remains the subject of active debate. Data from the past decade indicate that MPUC retains sensitivity to cisplatin-based chemotherapy, as evidenced by pathological complete response (pCR) rates comparable to those observed in conventional urothelial carcinoma [15,16]. At the same time, most studies fail to demonstrate a consistent improvement in overall survival with NAC, suggesting that pathological response does not fully compensate for the aggressive biological behavior of MPUC [11,13].

An increasing body of evidence indicates that the biological underpinnings of MPUC play a central role in its aggressive clinical phenotype. Molecular studies have shown that MPUC is characterized by a luminal phenotype, frequent ERBB2/HER2 overexpression, and activation of pathways associated with epithelial–mesenchymal transition (EMT) and angiogenesis [21,22]. Notably, transcriptomic analyses of high-grade T1 MPUC demonstrate that, even at an early clinical stage, its molecular profile resembles that of MIBC, providing a biological explanation for the observed propensity toward early progression and frequent upstaging [22]. While the present review focuses on bladder MPUC, it is important to acknowledge that certain molecular observations derive from studies of micropapillary carcinoma arising in the upper urinary tract. Given the shared urothelial lineage and overlapping morphological characteristics, such findings may provide valuable biological insight. However, anatomical differences in tumor microenvironment, patterns of dissemination, and surgical management warrant cautious interpretation. Consequently, translational parallels should not be construed as evidence of direct clinical equivalence across urothelial sites.

From a clinical standpoint, these data lead to several consistent conclusions. First, MPUC should be regarded as a distinct biological entity rather than merely a morphological variant of conventional urothelial carcinoma. Second, standard NMIBC treatment algorithms, including TURBT followed by intravesical BCG, are inadequate for a substantial proportion of patients with high-grade cT1 MPUC, as demonstrated by multiple retrospective studies published since 2015 [11,12,17]. Third, decisions regarding NAC should be individualized and cannot substitute for early radical treatment in high-risk patients.

Recent high-level evidence has further refined the interpretation of micropapillary differentiation in urothelial carcinoma. In particular, the contemporary analysis published by Bizzarri et al. [34] provides additional insight into the prognostic implications and therapeutic considerations associated with variant histology. The study highlights that while micropapillary features are consistently associated with adverse pathological characteristics—such as higher rates of lymphovascular invasion and nodal involvement—the independent prognostic effect beyond established determinants including pathological stage and lymph node status remains nuanced. Importantly, the authors emphasize the need for careful integration of histological subtype into multimodal treatment planning rather than its use as an isolated decision-making determinant. These findings align with our synthesis, supporting the concept that MPUC represents a marker of aggressive tumor biology that warrants intensified clinical vigilance, yet requires interpretation within the broader framework of stage, nodal status, and individualized risk assessment.

In summary, despite the growing body of literature published after 2015, the available evidence remains affected by substantial methodological heterogeneity. This underscores the need for standardized reporting of histological variants, prospective multicenter studies, and integration of clinical and molecular data. Only such an approach will allow a definitive determination of the extent to which micropapillary morphology constitutes an independent prognostic factor and will identify the patient subgroups most likely to benefit from intensified therapeutic strategies. The high probability of clinical understaging should be regarded as a key modifier of treatment algorithms and may justify deviation from standard NMIBC pathways in selected patients.

An additional unresolved issue concerns the role of immune checkpoint inhibitors in MPUC. Although ICIs are now standard in advanced urothelial carcinoma, variant histologies were not systematically analyzed in pivotal phase III trials. Consequently, therapeutic recommendations for MPUC in the metastatic setting are largely extrapolated from conventional urothelial carcinoma data. Given the aggressive phenotype and frequent advanced stage at presentation, inclusion of variant histologies in future prospective immunotherapy trials should be considered a research priority.

## 11. Conclusions

The micropapillary variant of bladder cancer represents a biologically and clinically distinct entity associated with a clearly unfavorable prognosis. Evidence derived from meta-analyses and large cohort studies consistently supports an early radical cystectomy strategy in patients with high-risk NMIBC MPUC, as well as consideration of neoadjuvant chemotherapy in the muscle-invasive setting. Ongoing advances in the understanding of the molecular underpinnings of MPUC offer promising opportunities for treatment personalization and the future integration of targeted therapeutic approaches. From a clinical perspective, early treatment intensification should be strongly considered whenever a micropapillary component is identified. Beyond summarizing existing evidence, the present review highlights persistent methodological gaps that limit definitive conclusions regarding independent prognostic impact. By proposing structured reporting standards and emphasizing the clinical implications of staging discrepancies, this work aims to facilitate more reliable future research and improved therapeutic decision-making in MPUC. The emerging role of immune checkpoint inhibitors in advanced urothelial carcinoma further underscores the need for variant-specific data to determine whether micropapillary differentiation modifies immunotherapy responsiveness.

## Figures and Tables

**Table 1:** Comparison of key studies on micropapillary variant of urinary bladder cancer (MPUC).

Reference	Study Type	The Number of Patients (MPUC)	Disease Stage	Treatment/Intervention	Primary Endpoints	Main Prognostic and Clinical Outcomes
Abufaraj et al. [11]	Meta-analysis (systematic review)	>1400 (together)	NMIBC + MIBC	Different strategies (TURBT, RC, NAC)	OS, CSS, PFS	MPUC significantly worsens OS and CSS; higher pT, more frequent N+; high progression rate with conservative treatment
Sui et al. [12]	Retrospective, NCDB	869	All	RC ± NAC	OS	Even a small micropapillary component → worse OS; more frequent ≥pT3 and N+
Mitra et al. [13]	Retrospective, multicenter	100	MIBC	RC ± NAC	OS, CSS	MPUC is an independent factor of poor prognosis after RC; high N+ rate
Jin et al. [14]	Retrospective	128	NMIBC + MIBC	RC/conservative treatment	OS, CSS	MPUC associated with shorter OS and CSS; stage and N status key predictors
Diamantopoulos et al. [15]	Retrospective	46	MIBC	NAC + RC	pCR, OS	Response to NAC comparable to classic UC; no clear improvement in OS
Rahman et al. [16]	Retrospective, multicenter	72	MIBC	NAC + RC vs. RC	pCR, OS	NAC enables pCR in some patients; survival benefit depends on patient selection.
Chou et al. [17]	Retrospective	73	Mainly NMIBC	TURBT ± RC	OS, PFS	High risk of progression in T1 HG; authors recommend early RC
Jojima et al. [18]	Retrospective	54	MIBC	RC	OS, RFS	High incidence of N+ (~40%); early relapses despite radical treatment
Liu et al. [19]	Retrospective	118	NMIBC + MIBC	Different	OS, CSS (nomograms)	pT stage, N status and MPUC component crucial for survival prediction
Kılıç et al. [20]	Retrospective	41	Different	Different	OS	Histological variants, including MPUC, significantly worsen the prognosis.
Guo et al. [21] ^#^	Molecular (gene expression)	31	Different	—	Molecular profile	ERBB2/HER2 overexpression, luminal signatures, EMT, aggressive biology
Bowden et al. [22]	Transcriptomic	23	T1 HG	TURBT	Gene signature	T1 MPUC shows a molecular profile similar to MIBC

MPUC—micropapillary urothelial carcinoma; NMIBC—non-muscle-invasive bladder cancer; MIBC—muscle-invasive bladder cancer; RC—radical cystectomy; NAC—neoadjuvant chemotherapy; OS—overall survival; CSS—cancer-specific survival; PFS—progression-free survival; RFS—recurrence-free survival; pCR—pathological complete response. ^#^ One included study [21] evaluated micropapillary carcinoma of the upper urinary tract and was incorporated solely for its molecular and therapeutic relevance (e.g., HER2 expression). This study was not used to support bladder-specific clinical outcome conclusions. Upper urinary tract cohort included for translational context only. → means implication.

**Table 2:** Neoadjuvant chemotherapy (NAC) vs. no NAC in patients with micropapillary variant of bladder cancer (MPUC).

Reference	Study Type	The Number of Patients (MPUC)	Stage	Groups Compared	Pathological Response	Survival Results	Main Clinical Findings
Sui et al. [12]	Retrospective, NCDB	869	MIBC	NAC + RC vs. RC	pCR ~10–15%	No clear OS improvement	NAC is rarely used; aggressive biologics for MPUC
Mitra et al. [13]	Retrospective	100	MIBC	NAC + RC vs. RC	Not reported	OS, CSS no significant difference	NAC does not eliminate the poor prognosis of MPUC
Diamantopoulos et al. [15]	Retrospective	46	MIBC	NAC + RC	pCR comparable to UC NOS	OS no significant improvement	MPUC is chemosensitive but biologically aggressive
Rahman et al. [16]	Multicenter, retrospective	72	MIBC	NAC + RC vs. RC	pCR in a subgroup	Trend towards OS improvement	Patient selection is key to the benefits of NAC
Abufaraj et al. [11]	Meta-analysis	>1400	NMIBC + MIBC	NAC + RC vs. RC	Variable	No consistent OS benefit	Early RC is more important than NAC
Chou et al. [17]	Retrospective	73	Mainly NMIBC	NAC occasionally	—	High progression	NAC does not replace early RC
Jojima et al. [18]	Retrospective	54	MIBC	RC ± NAC	Not reported	High risk of relapse	NAC does not eliminate the risk of N+

**Table 3:** The importance of clinical tumor stage (Clinical T stage) in micropapillary variant of bladder cancer (MPUC).

Reference	Study Type	The Number of Patients (MPUC)	Clinical T Stage	Predominant Management	Key Clinical Findings	Conclusions on the Significance of T Stage
Sui et al. [12]	Retrospective, NCDB	869	cTa–cT4	RC ± NAC	Frequent underestimation of clinical stage	High upstaging rate from cT1/cT2 to ≥pT3
Abufaraj et al. [11]	Meta-analysis	>1400	cT1–cT4	Varied	Poorer OS and CSS at each stage	Clinical T stage less accurately reflects the aggressiveness of MPUC
Mitra et al. [13]	Multicenter	100	cT2–cT4	RC ± NAC	Shorter OS even with low cT	cT2 MPUC behaves like more advanced UC
Jin et al. [14]	Retrospective	128	cT1–cT4	RC/conservative treatment	cT stage strongly correlates with OS	cT1 MPUC has a high risk of progression
Chou et al. [17]	Retrospective	73	Mainly cT1	TURBT ± RC	High rate of progression to MIBC	cT1 HG MPUC → early RC recommended
Jojima et al. [18]	Retrospective	54	cT2–cT3	RC	Frequent pT3/pT4 after RC	cT underestimates true stage
Liu et al. [19]	Retrospective (nomograms)	118	cT1–cT4	Different	cT is an independent predictor of OS	Combining cT with N and %MPUC improves prediction

→ means implication.

**Table 4:** The importance of pathological tumor stage (pT) in micropapillary variant of bladder cancer (MPUC).

Reference	Study Type	The Number of Patients (MPUC)	Pathological T Stage	Treatment	Key Findings	Prognostic Conclusions
Sui et al. [12]	Retrospective, NCDB	869	pT1–pT4	RC ± NAC	≥pT3 in >50% of patients	pT3–pT4 strongly associated with poorer OS
Abufaraj et al. [11]	Meta-analysis	>1400	pT1–pT4	Different	High percentage of pT3–pT4	pT stage a key determinant of OS and CSS
Mitra et al. [13]	Multicenter	100	pT2–pT4	RC	49% ≥pT3; high N+	pT and N independent risk factors
Jin et al. [14]	Retrospective	128	pT1–pT4	RC/conservative	pT ≥3 worsens OS and CSS	pT more important than cT for prognosis
Chou et al. [17]	Retrospective	73	pT1–pT4	TURBT ± RC	Frequent upstaging	pT reveals biological aggressiveness
Jojima et al. [18]	Retrospective	54	pT2–pT4	RC	pT3–pT4 in ~60%	Advanced pT → early relapses

→ means implication.

**Table 5:** Clinical T stage vs. Pathological T stage (upstaging matrix) in MPUC.

Clinical T Stage	Most Common Post-Cystectomy pT	Estimated Upstaging Rate	Clinical Significance
cT1	pT2–pT4	40–60%	High risk of progression; consider upfront RC
cT2	pT3–pT4	30–50%	Behavior similar to advanced MIBC
cT3	pT3–pT4	10–20%	Stage usually confirmed
cT4	pT4	<10%	Advanced systemic disease

**Table 6:** Management of patients with cT1 MPUC: BCG vs. upfront radical cystectomy (RC).

Reference	Study Type	The Number of Patients (cT1 MPUC)	Strategies Compared	Oncological Outcomes	Main Findings
Abufaraj et al. [11]	Meta-analysis	—	BCG vs. RC	High progression after BCG	RC preferred in cT1 HG MPUC
Sui et al. [12]	NCDB	201	BCG vs. RC	Better OS after RC	Conservative treatment carries risks
Chou et al. [17]	Retrospective	73	TURBT + BCG vs. RC	Early progression after BCG	Authors recommend upfront RC
Jin et al. [14]	Retrospective	52	Conservative vs. RC	RC improves CSS	cT1 MPUC ≠ classic NMIBC
Kılıç et al. [20]	Retrospective	41	Various	Variants worsen OS	Early RC justified

## Data Availability

No new data were created or analyzed in this study. Data sharing is not applicable to this article.

## Abbreviations

The following abbreviations are used in this manuscript:

MPUC	Micropapillary urothelial carcinoma
UC	Urothelial carcinoma
HR	Hazard ratio
NMIBC	Non-muscle-invasive bladder cancer
MIBC	Muscle-invasive bladder cancer
TURBT	Transurethral resection of the bladder tumor
RC	Radical cystectomy
NAC	Neoadjuvant chemotherapy
OS	Overall survival
CSS	Cancer-specific survival
PFS	Progression-free survival
RFS	Recurrence-free survival
pCR	Pathological complete response
BCG	Bacillus Calmette–Guérin
NCDB	National Cancer Database
